# Efficacy and Safety of Punch Excision Combined With Adjuvant Therapies for Hypertrophic Scars and Keloids: A Narrative Review

**DOI:** 10.1111/jocd.70622

**Published:** 2026-01-19

**Authors:** Qiang Fang, Xin Lin, Wen Liang, Weiming Qiu

**Affiliations:** ^1^ Department of Burn and Plastic Surgery General Hospital of Central Theater Command Wuhan China

## Abstract

**Background:**

Pathological scars manifest as firm, elevated, erythematous plaques, or nodules after skin injury. As challenging wound‐healing complications, hypertrophic scars and keloids significantly compromise aesthetics while causing functional impairment and psychosocial distress. Consequently, their management remains a central focus in dermatology. The skin punch has undergone substantial technical advancements, emerging as a promising therapeutic modality.

**Methods:**

To evaluate the efficacy of punch‐based techniques in managing pathological scars, we conducted a comprehensive literature search using Boolean logic across electronic databases, with the core search strategy built upon the following terms: (“skin punch” OR “punch excision” OR “punch volume reduction”) AND (“pathological scar” OR “hypertrophic scar” OR “keloid”). Additional relevant studies were identified through citation tracking of the retrieved articles. This review systematically examines recent advancements in punch‐assisted scar management, with particular focus on the mechanistic basis, diverse therapeutic modalities, clinical efficacy, and associated complications.

**Results:**

This systematic appraisal of punch volume reduction for pathological scars affirms its robust efficacy in restoring aesthetic contour and functional integrity, while concurrently revealing substantial interpatient heterogeneity in therapeutic response and protracted convalescence intervals. Although the technique's clinical value is well‐established, a critical deficit persists in stratified evidence across scar phenotypic spectra and temporal disease dynamics, with no codified protocols to direct therapeutic application. Confronted by the profound quality‐of‐life impairment attributable to pathological scars and accelerating clinical necessities, forthcoming research mandates large‐scale prospective cohorts with prolonged surveillance, emphasizing technical innovation, preemptive complication management, and individualized treatment algorithms. The consolidated insights presented herein deliver both actionable guidance for contemporary evidence‐based practice and a foundational conceptual architecture for future technological evolution in scar therapeutics.

## 1 Introduction

The human organism possesses an inherent capacity for adaptive regeneration following injury [1]. However, excessive reparative processes can trigger the development of pathological scars—notably hypertrophic scars and keloids—which represent dysregulated healing phenotypes. Cutaneous wound‐healing progresses through four sequential phases: hemostasis, inflammation, proliferation, and remodeling [2]. A pivotal mechanism in scar pathogenesis is the hyperproliferation of fibroblasts and dysregulated collagen metabolism during the proliferative phase [1]. Aberrant repair—characterized by persistent inflammation, heightened vascular density, and excessive collagen deposition—often culminates in pathological scarring. These lesions typically manifest as abnormal dermal hyperplasia, pruritus, and even functional impairment [3]. Harn et al. [4] have posited that hypertrophic scars and keloids represent phenotypic variants within a spectrum of a shared inflammatory fibroproliferative disorder. This pathological spectrum is modulated by the intensity and chronicity of inflammation in conjunction with mechanical stress—a paradigm substantiated by accumulating clinicopathological evidence [5]. The aberrant scar growth is driven by a chronic inflammatory response originating in the reticular dermis, triggered by diverse forms of cutaneous insult such as trauma, burns, surgical interventions, vaccination, dermal penetration, acne vulgaris, and herpes zoster infection [6]. Mechanical force serves not merely as a promoter of pathological scar growth but as an essential etiological factor in their pathogenesis. This is corroborated by the pronounced predisposition of pathological scars, particularly keloids, to develop in regions of high cutaneous tension [7]. Increased mechanical tension is hypothesized to disrupt normal granulation tissue remodeling [8], while concomitantly stimulating fibroblast proliferation and promoting the synthesis and aberrant accumulation of collagen fibers through the dysregulation of profibrotic protein expression [9]. Furthermore, elevated skin tension enhances extracellular matrix (ECM) deposition and amplifies cellular contractility, mechanisms that collectively contribute to scar progression and persistence [10].

The primary therapeutic objectives in the management of pathological scars are the reduction of scar volume [11], improvement of aesthetic appearance, alleviation of patient‐reported symptoms (notably pain and pruritus), and correction of associated functional impairments [12, 13]. Contemporary clinical practice emphasizes a multimodal strategy, combining surgical excision with a range of nonsurgical modalities—such as intralesional pharmacotherapy, silicone‐based sheeting, laser and energy‐based devices, microneedling, and radiofrequency techniques—to optimize outcomes and minimize the risk of recurrence [14]. Nonetheless, recurrence rates, particularly following nonsurgical management of keloids, remain unacceptably high. Treatment efficacy is further governed by a complex interplay of determinants, including the underlying etiology, specific scar phenotype, local biomechanical tension, lesion size and multiplicity, patient‐specific socioeconomic factors, and available healthcare resources [15]. Consequently, the development of more effective and durable therapeutic protocols for pathological scarring continues to represent a substantial clinical challenge in dermatologic and plastic surgery [8].

Surgical excision, which functions to mitigate mechanical tension at the keloid periphery and attenuate local wound inflammation [16], represents a conventional yet expeditious modality for keloid removal [17]. It is specifically indicated in cases of functional impairment or severe contracture [18]. Substantial clinical evidence corroborates the current consensus that a combined regimen of surgical excision and adjuvant radiotherapy constitutes the optimal therapeutic strategy for pathological scars [19]. However, for specific clinical subtypes—including recurrent lesions following previous excision and non‐resectable cases characterized by extensive surface area, distal limb localization, sites with limited tissue availability under high tension, or inadequate response to systemic/topical corticosteroids and laser therapy [20]—intralesional (core) excision or partial resection is recommended as the preferred surgical alternative [21, 22]. Within this clinical context, punch excision has rapidly emerged as a technique of significant interest in scar management. In contrast to extensive surgical resection, punch excision is distinguished by its procedural simplicity, capacity for suture‐free closure, and avoidance of secondary wound creation [23]. This approach effectively reduces the wound burden, accelerates healing, and enhances procedural efficiency, thereby facilitating its increasing integration into clinical practice [11].

## 2 Punch Excision

### 2.1 Operative Protocol for Punch Excision

**FIGURE 1:**
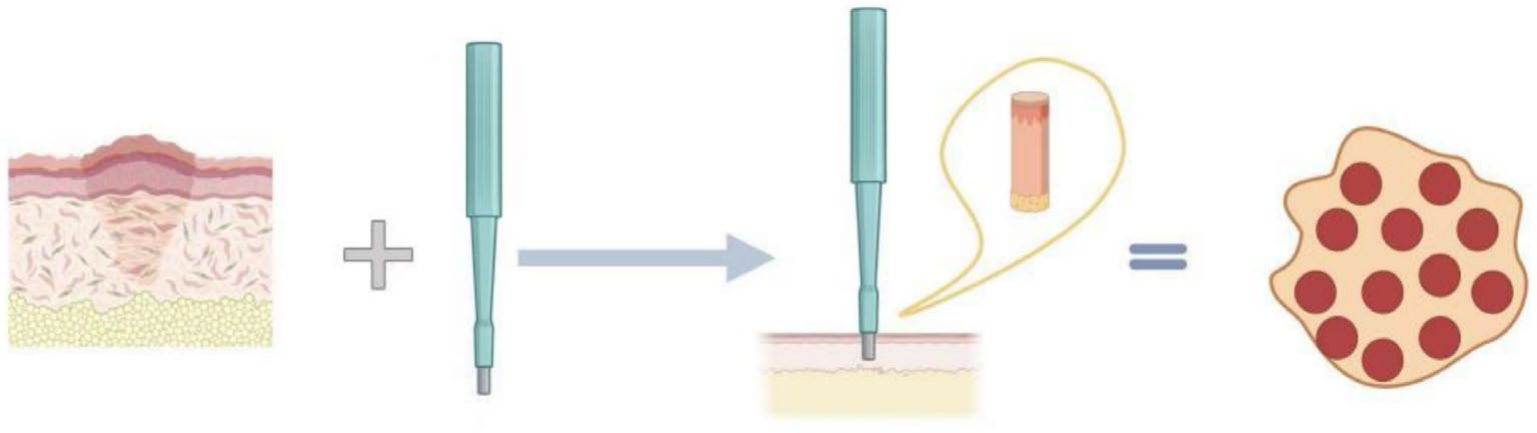
Schematic of the punch excision procedure for pathological scars. The schematic depicts the technique from scar assessment to postoperative outcome: pathological scar tissue architecture and instrument selection based on morphology; surgical execution via perpendicular alignment, rotational advancement through the pathological core, and full‐thickness resection to the superficial adipose layer (inset: excised specimen); and the immediate postoperative wound with geometrically optimized punch distribution (inter‐site spacing equal to instrument diameter) and suture‐free closure achieved through biomechanical redistribution of tension vectors and tissue laxity recruitment.

The surgical site is preoperatively demarcated to ensure precise localization and orientation for the planned punch excisions. Following aseptic preparation and draping, local anesthesia is administered through layered perilesional and intralesional infiltration. A cylindrical punch is then advanced through the pathological scar tissue using a vertical, rotational motion to achieve an en bloc excision of the scar core (Figure 1) [24]. An inter‐punch distance equivalent to the instrument's diameter is typically maintained [25]. While a reduced inter‐punch distance enhances the volume of scar tissue removal, excessively narrow spacing may compromise microvascular perfusion, potentially cascading into localized necrosis and ulceration. Conversely, overly conservative spacing leaves behind substantial residual scar tissue, resulting in suboptimal clinical outcomes. Therefore, determining the physiologically optimal inter‐punch distance is critical. Luo et al. [23] recommend a spacing of approximately 2 mm based on clinical observations, whereas Luo et al. advocate for a narrower interval of about 0.5 mm [24]. In cases where residual scar tissue persists at the lesion periphery following initial excision, supplementary tangential punch excisions—oriented parallel to the skin surface along the scar margin—may be performed. The deepest portion of the subcutaneous scar base is then carefully undermined and transected using tissue forceps and curved dissecting scissors. Finally, the wound is managed with a sterile pressure dressing [26].

### 2.2 Punch Excision Mechanics

Punch excision facilitates substantial physical debulking by directly extracting core components of pathological scar tissue, thereby generating a perforated, microcylinder‐based architecture within the lesion. This strategically engineered microstructure promotes uniform distribution of biomechanical loads, enhancing both tissue pliability and viscoelastic compliance [27]. The subsequent attenuation of focal tensile stress creates a mechanobiological environment conducive to collagen fiber reorganization and the regeneration of physiologically functional dermal tissue, ultimately leading to progressive flattening of the scar surface. In comparison to conventional excision, punch excision utilizing devices with diameters below 3 mm exhibits a distinctly superior clinical profile, characterized by a marked reduction in complications such as infection, hemorrhage, and flap necrosis; a propensity for favorable healing by secondary intention without the need for sutures; a diminished risk of recurrent or new‐onset pathological scarring; aesthetically refined outcomes; and consequently, higher patient‐reported satisfaction scores [28].

In a prospective comparative study with a 3‐year follow‐up, Chen et al. demonstrated that punch excision (*n* = 40) yielded superior outcomes compared to a control cohort managed with conventional nonsurgical modalities (*n* = 40), including pharmacotherapy, silicone‐based therapy, laser treatment, microneedling, and radiofrequency. The intervention group exhibited significantly accelerated wound healing alongside notable improvements across key scar parameters—specifically, reductions in erythema, vertical height, and horizontal width, accompanied by enhanced tissue pliability [15]. Moreover, the punch excision approach substantially shortened the overall treatment duration and provided more effective symptomatic relief from pain and pruritus. These findings are corroborated by a separate clinical investigation focusing specifically on keloids, which established the sustained long‐term superiority of punch excision over non‐punch therapies, such as pharmaceutical interventions, intralesional injections, and laser treatment [26]. From a mechanistic perspective, the microconduits created by punch excision enable deep and homogeneous intralesional dispersion of topical agents when applied postoperatively. This markedly enhances transdermal delivery efficiency and promotes more uniform collagen remodeling, ultimately facilitating superior restoration of skin surface planarity [23].

### 2.3 Contraindications and Complications of Punch Excision

In a systematic clinical analysis of 1294 patients (540 males, 754 females), Yasui et al. characterized the complication profile and identified risk factors associated with punch excision procedures. The most prevalent adverse events documented were localized postoperative hemorrhage (0.9%), surgical site infection (0.2%), iatrogenic injury to adjacent skin (0.2%), and vasovagal reflex (0.1%) [29]. A comprehensive preoperative evaluation is mandatory, encompassing screening for underlying coagulopathies and a meticulous review of medications, with special consideration given to anticoagulants and antiplatelet agents that may disrupt hemostasis. Moreover, any documented hypersensitivity to local anesthetics, antiseptic preparations, topical antibiotics, or medical adhesives warrants the implementation of alternative clinical protocols [30]. From a technical standpoint, the complete removal of scar tissue is critical. Any residual tissue fragments must be meticulously extracted using serrated forceps to prevent the development of implantation cysts and subsequent diminution of therapeutic efficacy [31].

## 3 Combination Therapy With Punch Excision

Current clinical evidence suggests that the volumetric reduction achieved through punch excision, while effective for scar debulking, concomitantly induces a phenotypic shift of fibroblasts into a hyperproliferative state and exacerbates the local inflammatory cascade [32]. As a result, monotherapy with punch excision is frequently inadequate for durable recurrence prevention, underscoring the necessity for integrated [27], multimodal treatment strategies to optimize long‐term patient‐reported outcomes.

### 3.1 Combination Therapy With Pressure Treatment

Pressure therapy is well‐established as a primary prophylactic and adjuvant intervention for hypertrophic scarring [33], although its molecular mechanisms remain incompletely delineated. De Decker et al. [34] posit that pressure‐induced tissue hypoxia downregulates transforming growth factor‐beta 1 (TGF‐β1) expression, thereby attenuating fibroblast activation. Research by Cao et al. further indicates that elevated mechanical loading impairs tissue perfusion and oxygen delivery, promoting apoptosis, stabilizing mast cells, and consequently suppressing collagen synthesis while facilitating collagen realignment. This cascade also inhibits angiogenesis and reduces scar firmness [5]. Bailey et al. [35] demonstrated that compression garments maintaining 15–25 mmHg pressure elicit localized vasoconstriction, which reduces inflammatory cytokine release and subsequently diminishes collagen deposition, scar formation, thickness, induration, and erythema. Clinical guidelines, such as those summarized by Ojeh et al. [36], recommend initiating pressure therapy (20–40 mmHg) for 12–23 h daily within the early re‐epithelialization phase, continuing for over 6 months to prevent pathological scarring. Nevertheless, therapeutic efficacy is substantially limited by patient compliance, often compromised by wearing discomfort [37]. Moreover, numerous clinical evaluations confirm that pressure monotherapy achieves only modest improvements in scar thickness and pruritus, generally falling short of patient expectations for cosmetic and functional outcomes [38, 39]. In support, Anderson et al. reported a 5% recurrence rate following keloid excision coupled with 18 months of postoperative pressure therapy. Therefore, pressure therapy is most effectively implemented as an integral component of a multimodal management strategy for pathological scars [18].

### 3.2 Combination Therapy With Intralesional Corticosteroid Injections and Adjunctive Agents

Intralesional pharmacotherapy represents a cornerstone in the management of pathological scars, primarily categorized into corticosteroid‐based interventions—recognized as the gold standard—and non‐corticosteroid agents, including verapamil hydrochloride, 5‐fluorouracil (5‐FU), bleomycin, interferon‐α2b, pentoxifylline, botulinum toxin type A (BTA), hyaluronidase, and platelet‐rich plasma (PRP) [15, 36]. The therapeutic primacy of corticosteroids stems from their pleiotropic mechanisms of action. As elucidated by Frech et al., they effectively suppress fibroblast proliferation by inducing apoptotic pathways and downregulating transforming growth factor‐β1 (TGF‐β1) expression, while simultaneously impairing angiogenesis through disruption of vascular endothelial growth factor (VEGF) and α‐globulin‐mediated signaling. Complementing these findings [8]. Gladko and Izmaylova [40] have demonstrated that corticosteroids further confer immunomodulatory benefits via lymphocyte inhibition and subsequent reduction in the synthesis and bioactivity of key pro‐inflammatory cytokines. Among these agents, triamcinolone acetonide (TAC) remains the most extensively employed formulation in clinical settings. Intralesional TAC administration has been robustly validated to significantly diminish scar volume and height, improve tissue pliability, alleviate concomitant pain and pruritus, and reduce recurrence rates [41]. Non‐corticosteroid agents, by contrast, operate through distinct pathways: 5‐FU, a fluorinated pyrimidine analog, acts by inhibiting DNA synthesis, thereby curtailing fibroblast expansion and collagen deposition [42]; both bleomycin and interferon‐α2b function to suppress collagen production and fibroblast proliferation; pentoxifylline exhibits marked anti‐inflammatory and antifibrotic activities; verapamil modulates extracellular matrix remodeling by reducing collagen biosynthesis while promoting collagenase expression; PRP enhances extracellular matrix collagen synthesis [43]; and botulinum toxin type A induces localized chemodenervation, thereby reducing mechanical tension and shortening the inflammatory phase in wound healing [44].

The inherently dense and hypovascular nature of pathological scar tissue fundamentally limits homogeneous drug dispersion during intralesional delivery, often resulting in subtherapeutic depot formation and compromised biodistribution [45]. While intralesional corticosteroid monotherapy remains a cornerstone intervention, its efficacy is highly variable, with reported response rates of 50%–100% juxtaposed against concerning recurrence rates of 9%–50%. This therapeutic modality is further constrained by a nontrivial profile of local adversities, including procedure‐associated pain, pigmentary dysregulation (encompassing both hyperpigmentation and hypopigmentation), telangiectasia, dermal atrophy, ulceration, and in select cases, paradoxical scar worsening [46]. These limitations are compounded by the absence of standardized protocols governing critical parameters such as optimal dosing, injection frequency, and treatment intervals, fostering substantial heterogeneity in clinical practice [15]. To holistically address these challenges, multimodal treatment strategies are strongly advocated to synergistically enhance therapeutic outcomes, maximize patient adherence, and mitigate adverse event risks. Validating this paradigm, Choi et al. [47] documented a markedly reduced recurrence rate of merely 5% during a 24‐month follow‐up period in a cohort of 20 patients with auricular keloids managed via punch excision coupled with intralesional triamcinolone acetonide administration.

### 3.3 Combination Therapy With Radiotherapy

Radiotherapy represents a cornerstone intervention in both the management and prevention of pathological scarring [35]. As a monotherapy, it effectively alleviates pain and pruritus while improving scar pigmentation and thickness; however, its utility is limited by a substantial recurrence rate of approximately 37%. When deployed as an adjuvant to surgical excision within the critical 24‐h postoperative window—a period characterized by maximal radiosensitivity of proliferating fibroblasts and nascent microvasculature—recurrence rates are markedly reduced to approximately 15% [48, 49]. The therapeutic mechanism operates through a dual pathway: direct cytostatic effects via the induction of apoptosis and suppression of fibroblast hyperproliferation, combined with anti‐angiogenic actions and immunomodulation through the inhibition of lymphocyte and macrophage activation, thereby disrupting the cycle of persistent inflammation [50]. Several radiotherapeutic techniques have been successfully utilized following excision, including electron beam radiotherapy, brachytherapy, superficial X‐ray, and orthovoltage therapy [36]. Among these, electron beam irradiation, with its tunable tissue penetration of 2–6 cm, is particularly suited for cutaneous targeting [18]. Comparative efficacy analysis establishes brachytherapy as the most effective modality (15% recurrence), followed by both X‐ray and electron beam techniques (23%). The adverse event profile is dominated by cutaneous reactions, with pigmentary changes—ranging from erythema and transient hyperpigmentation to persistent dyspigmentation—alongside ulceration and desquamation, occurring in approximately 32.5% of cases [51]. These manifestations are typically manageable with topical emollients or corticosteroid‐based formulations. Paramount to treatment planning is the consideration of radiation‐induced carcinogenesis risk, particularly concerning irradiation of critical structures and exposure from scatter radiation. Consequently, stringent patient selection is mandatory, with extreme caution advised for radiation‐sensitive regions such as the head, neck, thyroid, and breast, and contraindication in pediatric patients under 18 years of age [36].

### 3.4 Combination With Laser and Light‐Based Therapies

Pathological scarring is fundamentally driven by aberrant microvascular proliferation and dysregulated collagen metabolism, clinically presenting as persistent erythema and substantial tissue induration [52]. Energy‐based devices offer a targeted therapeutic approach that directly addresses these pathological components through minimally or noninvasive modalities characterized by excellent patient tolerability, demonstrated efficacy, abbreviated recovery, and a favorable safety profile [53]. The capacity for large‐area treatment per session further establishes these technologies as synergistic elements within multimodal scar management protocols (Table 1) [56]. Laser systems, operating on the principle of selective photothermolysis, are categorically divided into ablative and non‐ablative types. Established ablative platforms include the 2940 nm Erbium:Yttrium‐Aluminum‐Garnet (Er:YAG) and 10 600 nm carbon dioxide (CO_2_) lasers, which achieve their effects through precise energy absorption by tissue water, resulting in controlled vaporization and volumetric reduction [54]. Fractional CO_2_ laser technology exemplifies this approach by creating microscopic thermal treatment zones (MTZs) within a framework of viable tissue, thereby stimulating neocollagenesis and organized tissue regeneration [3]. In contrast, non‐ablative laser systems—including the 1550 nm erbium‐doped fiber laser, 1927 nm thulium fiber laser, 585/595 nm pulsed dye lasers (PDL), and 1064 nm Neodymium:Yttrium‐Aluminum‐Garnet (Nd:YAG) laser—function through selective photothermolysis of oxyhemoglobin, inducing precise microvascular injury, thrombosis, and transient ischemia [57]. This cascade promotes collagen denaturation, fiber reorganization, and reduced type III collagen deposition, collectively manifesting as clinical improvement in scar thickness, pigmentation, and vascularity [58]. The critical distinction between these modalities lies in their tissue interaction: while ablative lasers achieve their effect through epidermal ablation—carrying inherent risks of dyspigmentation and scarring with prolonged recovery—non‐ablative lasers selectively coagulate dermal targets while maintaining epidermal integrity, thereby offering superior safety characteristics [54]. In contrast to the spatially coherent, monochromatic emission characteristic of laser systems, intense pulsed light (IPL) generates noncoherent, polychromatic radiation across a broad spectrum (400–1200 nm). The subsequent development of narrow‐spectrum IPL, designated dense pulsed light (DPL), represents a spectral optimization through confinement to a 500–600 nm window. This tailored bandwidth corresponds precisely to the dual absorption maxima of oxyhemoglobin at 542 and 577 nm, thereby maximizing selective photothermolysis of vascular components while maintaining structural integrity of the surrounding epidermis [53]. The DPL platform simultaneously engages two critical chromophores—oxyhemoglobin and melanin—inducing microvascular coagulation, facilitating melanin clearance, and promoting neocollagenesis. These coordinated biological processes collectively mediate symptomatic relief of pruritus and erythema in hypertrophic scars while exerting inhibitory effects on fibroblast hyperproliferation [55].

**TABLE 1:** Comparative analysis of common photoelectric modalities for pathological scarring.

Classification	Indications	Advantages	Limitations
2940 nm Er:YAG Laser 10 600 nm CO_2_ Laser	Er:YAG Laser (2940 nm): First‐line for superficial atrophic scars and delicate resurfacing, leveraging its minimal thermal footprint. CO_2_ Laser (10 600 nm): Preferred for volumetric scar reduction (e.g., hypertrophic scars/keloids), utilizing ablative‐coagulative synergy. Combined use optimizes efficacy: Er:YAG refinishes surface texture, while CO_2_ addresses deep scar architecture [54]	Reduction in lesion volume stimulates collagen remodeling and induces dermal regeneration [36]	Adverse reactions encompass severe/procedural pain, persistent erythema, hyperpigmentation, hypopigmentation, and scar recurrence. These events demonstrate elevated incidence in Fitzpatrick IV–VI skin types, necessitating precise parameter control with real‐time dosimetry adjustment [53]
1064 nm Nd:YAG Laser	Deep penetration ability (up to 2 mm), making it the preferred modality for treating deep dermal vascular lesions such as hypertrophic scars and keloids neovascularization [53]	Photoelectric therapies constitute minimally invasive or noninvasive interventions characterized by tolerable pain profiles, superior therapeutic efficacy, abbreviated recovery periods, and infrequent adverse events. A single treatment session enables large‐area coverage	Parameter optimization is imperative, particularly given that certain clinical devices lack integrated cooling systems. This deficiency heightens risks of intraepidermal vesiculation and necessitates stringent thermal damage prevention to mitigate paradoxical scar hypertrophy
PDL (585/595 nm)	Superficial penetration (up to 1.2 mm), reducing intra‐scar vascularity and improving tissue pliability [53]		Purgura manifestation occurs when smaller spot sizes confine photothermal effects to superficial scar strata, limiting penetration depth [3]
IPL/DPL	IPL/DPL are indicated for all pathological scar phenotypes, effectively addressing scar‐associated dyschromia and microvascular abnormalities through selective photothermolysis [53, 55]		Limited penetration depth necessitates combination laser therapy, with significantly elevated vesiculation incidence [53]

Notwithstanding these mechanistic advantages, energy‐based device monotherapy presents several clinically significant limitations across heterogeneous scar phenotypes. Chief among these is the operator‐dependent risk of iatrogenic scar exacerbation through suboptimal parameter selection. Secondly, the inherent constraints of optical penetration depth necessitate repetitive treatment sessions at 4–6 week intervals for substantial scar volumes, frequently requiring concurrent modalities to achieve meaningful dimensional reduction [59]. Third, substantial capital investment in device acquisition translates to prohibitive treatment costs, creating socioeconomic barriers that systematically compromise therapeutic adherence. Fourth, laser monotherapy modalities—notably pulsed dye laser (PDL), carbon dioxide (CO_2_), and neodymium‐doped yttrium aluminum garnet (Nd:YAG) systems—demonstrate substantial recurrence rates, with clinical recurrence typically manifesting within 6–24 months posttreatment [36]. Fifth, the adverse effect profile encompasses procedure‐related pain, persistent erythema, pigmentary disturbances (encompassing both hyperpigmentation and hypopigmentation), and scar recurrence. Consequently, adjunctive punch excision is therapeutically warranted [53]. The substantial tissue debulking achieved through this intervention creates structural conditions conducive to augmented energy delivery to deeper scar compartments, resulting in marked reduction of the treatment duration.

## 4 Conclusion

Given the constrained efficacy of conventional therapeutic regimens, punch excision emerges as a superior intervention for pathological scars, offering three cardinal benefits: (1) Precision Microdebridement: The circular blade geometry enables architecturally defined resection of scar tissue with quantifiable depth and diameter, effectively neutralizing pathological tension vectors while conserving peri‐scar healthy tissue and generating a linearized cutaneous contour with enhanced cosmesis; (2) Sustained Therapeutic Remodeling: Through complete extirpation of the fibrotic core, this technique instigates a durable reparative process that surpasses the transient biological effects of non‐ablative modalities, exhibiting prolonged clinical stability and reduced recurrence rates when supported by standardized postoperative care; (3) Enhanced Procedural Efficacy: The optimized surgical workflow ensures reproducible execution under local anesthesia with minimal operative time, consequently attenuating procedural trauma, expediting tissue recovery, and promoting rapid psychosocial reintegration. We posit that the principal merit of punch excision resides in its capacity to serve as a pivotal initiator and synergistic component within multimodal therapeutic frameworks. Accumulating evidence substantiates that combining punch excision with targeted adjuvant therapies (e.g., pharmacologic agents, radiotherapy, laser) leverages complementary mechanistic pathways—integrating physical tissue reduction with biological response modification. This paradigm not only circumvents the dose‐limiting toxicities inherent in monotherapy intensification for recurrence prevention, but also enables multipronged suppression of the complex pathophysiological network underlying pathological scarring. Consequently, such integrated approaches achieve fundamental reductions in recurrence rates through simultaneous modulation of biomechanical forces, inflammatory cascades, and fibroproliferative signaling pathways. It is by virtue of this robust mechanistic synergy that punch excision‐based combination strategies are gaining prominence in contemporary scar management protocols.

Looking ahead, the progressive deciphering of the molecular pathogenesis underlying pathological scars, along with continual technological innovations, is poised to steer the evolution of punch excision‐based combinatorial therapies toward truly precision‐based and individualized treatment frameworks. Our strategic research direction is committed to constructing a comprehensive evidence ecosystem that clarifies the mechanistic contributions of punch excision to scar restoration, thus paving the way for methodical clinical validation and successful translation into practice. These concerted advances are anticipated to culminate in superior therapeutic options that not only effect a fundamental decline in recurrence rates but also generate sustained, clinically meaningful enhancements in long‐term, scar‐specific patient‐reported outcomes, and quality of life.

## Author Contributions

Xin Lin and Qiang Fang made equally significant contributions to the reference collecting, figure drawing, and manuscript writing. Weiming Qiu and Wen Liang designed and wrote the manuscript.

## Ethics Statement

The authors have nothing to report.

## Conflicts of Interest

The authors declare no conflicts of interest.

## Data Availability

As a narrative review, this work synthesizes evidence exclusively from existing published literature. No new primary data were generated during this study. All supporting information is derived from the referenced sources available in the public domain, primarily sourced from PubMed/MEDLINE (National Library of Medicine, https://pubmed.ncbi.nlm.nih.gov) and China National Knowledge Infrastructure (CNKI, https://www.cnki.net).
